# Supplementary material for: From sequence to enzyme mechanism using multi-label machine learning
Source: BMC Bioinformatics. 2014 May 19;15:150. doi: 10.1186/1471-2105-15-150 (PMC4229970; doi:10.1186/1471-2105-15-150)
Supplement: Additional file 2 — Java code of ml2db. Additional file ml2db_code.tar.gz contains the Java source code to run the multi-label machine learning experiments and save the results to database. The code’s Javadoc is included. [file 1471-2105-15-150-S2.zip › additional file 2/ml2db/ml2db/doc/index-files/index-7.html]

G-Index


JavaScript is disabled on your browser.


- Overview
- Package
- Class
- Use
- Tree
- Deprecated
- Index
- Help

- Prev Letter
- Next Letter

- Frames
- No Frames

- All Classes

A B C D E F G H I J K L M N O P Q R S T U V W X 


## G

generateAllAttributeNamesString() - Method in class main.ml2db.mulan.mulanarff.IdInstance


generateAndWriteArffFile() - Method in class main.ml2db.weka.Arff
:   The core of the method to write Weka style ARFF files: save properties to
    experiment table, load data from data database and save arff to file

generateArff(String) - Method in class test.ml2db.mulan.attributesfilter.ArffGeneratorForFilterTests
:   Generate an arff file and return the arff record id

generateAttributesNamesString(Instance, Set<Attribute>) - Method in class main.ml2db.mulan.mulanarff.IdMultiLabelInstances
:   Generates a -- separated string with the name of all the non-zero valued
    attributes of an instance.

generateBinaryAttribute(String) - Method in class main.ml2db.weka.AttributeFactory
:   Generates a binary attribute

generateDataSet(int, DbManagers) - Static method in class main.ml2db.mulan.mulanarff.database.MulanArffInstancesLoader


generateDataSet(String, String, DbManagers) - Static method in class main.ml2db.mulan.mulanarff.database.MulanArffInstancesLoader


generateExperimentManager(int, UtilityMetric) - Static method in class test.ml2db.al.ExperimentDetailsTest


generateIdInstances(int, DbManagers) - Static method in class main.ml2db.mulan.mulanarff.database.MulanArffInstancesLoader


generateInstanceToFeaturesMap() - Method in class main.ml2db.al.utility.pickers.byfrequency.NearestNeighboursByFreqUtility


generateNominalAttribute(String, Vector<String>) - Method in class main.ml2db.weka.AttributeFactory
:   Generate a nominal attribute (with a list of possible values).

generateNominalAttributeWithDummy(String, Set<String>) - Method in class main.ml2db.weka.AttributeFactory
:   Generate a nominal attribute (with a list of possible values).

generateNonZeroAttributesNames() - Method in class main.ml2db.mulan.mulanarff.IdInstance


generateNonZeroFeatureAttributesOfTheseInstances() - Method in class main.ml2db.mulan.mulanarff.IdMultiLabelInstances
:   Get the non-zero valued feature attributes of these instances

generateNumericAttribute(String) - Method in class main.ml2db.weka.AttributeFactory
:   Generates a numeric attribute

generatePartition(IdInstance, int[], int) - Method in class main.ml2db.mulan.mulanarff.IdMultiLabelInstances
:   The boolean[] partition of the labels for this instance (true if the
    label is present, false if absent, for each of the existing labels).

generatePredictions(MultiLabelOutput, IdInstance, IdMultiLabelInstances, boolean[]) - Method in class main.ml2db.mulan.predict.PredictionsSaver


generateTestArff() - Method in class test.ml2db.mulan.attributesfilter.ArffGeneratorForFilterTests
:   Generate the old test arff and return the arff record id

generateTestArffAndReturnId(String) - Static method in class test.ml2db.mulan.mulanarff.MulanArffTest
:   Create test arff file

generateTrainArff() - Method in class test.ml2db.mulan.attributesfilter.ArffGeneratorForFilterTests
:   Generate the old train arff and return the arff record id

generateXmlFile(TreeSet<String>) - Method in class main.ml2db.mulan.mulanarff.MulanArff
:   Generate a hierarchical XML files of the classes

get(int) - Method in class main.ml2db.mulan.mulanarff.IdMultiLabelInstances
:   For test purposes only! slow!

get(String) - Method in class main.ml2db.mulan.mulanarff.IdMultiLabelInstances


getActiveLearner() - Method in class main.ml2db.al.learning.AlExperimenter


getActiveLearningTestsetArffWithNN() - Static method in class test.ml2db.al.utility.neighbourhood.NeighbourhoodExperimentDetailsTest


getActiveLearningTrainsetArff() - Static method in class test.ml2db.al.ActiveLearningTest


getActiveLearningTrainsetArffWithNN() - Static method in class test.ml2db.al.utility.neighbourhood.NeighbourhoodExperimentDetailsTest


getAlExperimenterUnlabTrainSetLabTrainSet() - Static method in class test.ml2db.al.AlExperimenterTest


getAlgorithm() - Method in class main.ml2db.al.learning.ExperimentDetails


getAlgorithmName() - Method in class main.ml2db.mulan.predict.PredictionsSaver


getAllAttributeNames() - Method in class main.ml2db.mulan.mulanarff.IdMultiLabelInstances


getAllAttributes() - Method in class main.ml2db.mulan.mulanarff.IdMultiLabelInstances


getALOnTestsetArffWithConfidenceMetric() - Static method in class test.ml2db.al.utility.confidence.ConfidenceMetricTest


getAlTestsetArffWithGlobalPositioning() - Static method in class test.ml2db.al.ActiveLearningTest


getAlTestsetArffWithGlobalPositioningAndConf() - Static method in class test.ml2db.al.ActiveLearningTest


getAlTestsetWithAttRandomPicker() - Static method in class test.ml2db.al.utility.pickers.RandomAttributePickerTest


getAlTestsetWithAttSetPickerByFrequency() - Static method in class test.ml2db.al.utility.pickers.AttSetByFrequencyPickerTest


getAlTestsetWithAttSetRandomPicker() - Static method in class test.ml2db.al.utility.pickers.RandomAttributeSetPickerTest


getAlTestsetWithGlobalPositionMetric() - Static method in class test.ml2db.al.utility.globaldistance.GlobalDistanceMetricTest


getAlTestsetWithGlobDistanceWithConfidenceMetric() - Static method in class test.ml2db.al.utility.hybrid.GlobalDistanceWithConfidenceMetricTest


getAlTestsetWithLabByFreqPicker() - Static method in class test.ml2db.al.utility.pickers.LabelsByFreqPickerTest


getAlTrainsetWithGlobalPositionMetric() - Static method in class test.ml2db.al.utility.globaldistance.GlobalDistanceMetricTest


getAnnotationsReader() - Static method in class test.ml2db.mulan.measuremetrics.AnnotationsReaderTest
:   Test method for
    `main.ml2db.mulan.measuremetrics.AnnotationsReader#AnnotationsReader(java.lang.String, java.lang.String, java.lang.String)`
    .

getArff(String) - Static method in class test.ml2db.weka.ArffTest


getArffAndResultsDbManagers() - Method in class main.ml2db.al.learning.ExperimentDetails


getArffDb() - Method in class main.ml2db.DbManagers


getArffDbConnPath() - Method in class main.ml2db.mulan.mulanarff.database.MulanArffRecord


getArffDbManager() - Static method in class test.ml2db.weka.ArffPropsTableTest


getArffFileName() - Method in class main.ml2db.mulan.mulanarff.database.MulanArffRecord


getArffFilePath() - Method in class main.ml2db.mulan.mulanarff.database.MulanArffRecord


getArffFilePath() - Method in class main.ml2db.mulan.mulanarff.IdMultiLabelInstances


getArffFilePath() - Method in class main.ml2db.weka.ArffProps
:   Get the path to the ARFF file

getArffFilePath() - Method in class main.ml2db.weka.ImportArff


getArffFilePathTrainFile() - Static method in class test.ml2db.mulan.mulanarff.MulanDataSetManagerTest


getArffGenerator() - Method in class main.ml2db.mulan.mulanarff.MulanDataSetManager


getArffGenerator() - Method in class main.ml2db.weka.DataSetManager


getArffId() - Method in class main.ml2db.mulan.mulanarff.database.MulanArffRecord


getArffId() - Method in class main.ml2db.mulan.mulanarff.IdMultiLabelInstances


getArffPath() - Method in class main.ml2db.weka.Arff


getArffPathFromDb(int) - Method in class main.ml2db.weka.ArffRecordsTable


getArffProperties() - Method in class main.ml2db.weka.DataSetChecker


getArffProperties() - Method in class main.ml2db.weka.DataSetManager


getArffProperties1() - Static method in class test.ml2db.dataharness.ArffPropsOneTest


getArffProperties2() - Static method in class test.ml2db.dataharness.ArffPropsTwoTest


getArffPropsPathFromDb(int) - Method in class main.ml2db.weka.ArffRecordsTable


getArffRealRestTestDbManagers() - Static method in class test.ml2db.dataharness.TestProjectParameters


getArffRecord() - Method in class main.ml2db.mulan.learn.MulanSerializer


getArffRecordDbMan() - Method in class main.ml2db.mulan.mulanarff.database.MulanArffRecord


getArffRecordId() - Method in class main.ml2db.weka.Arff


getArffTable() - Method in class main.ml2db.mulan.attributesfiltered.AttFilter


getArffWithNoXmlProperty() - Static method in class test.ml2db.weka.ArffTest


getArffWithNumericAttProperties() - Static method in class test.ml2db.weka.ArffTest


getAttributeAbsentOption() - Method in class main.ml2db.weka.AttributeFactory
:   private void generateZeros() { double[] zeros = new
    double[this.getAttributes().size()]; Arrays.fill(zeros, 0.0); m\_zeros =
    zeros;
    }

getAttributeByName(String) - Method in class main.ml2db.weka.AttributeFactory


getAttributeIndices() - Method in class main.ml2db.weka.JaccardDistanceStored


getAttributeNames() - Method in class main.ml2db.weka.InstAttValueMap


getAttributePresentOption() - Method in class main.ml2db.weka.AttributeFactory


getAttributes() - Method in class main.ml2db.weka.AttributeFactory


getAttributes() - Method in class main.ml2db.weka.DataSetGenerator


getAttributesArray() - Method in class main.ml2db.mulan.mulanarff.IdMultiLabelInstances


getAttributesFactory() - Method in class main.ml2db.mulan.mulanarff.MulanDataSetGenerator


getAttributesFactory() - Method in class main.ml2db.weka.DataSetGenerator


getAttributesFilter() - Static method in class test.ml2db.mulan.attributesfilter.AttFilterTest


getAttributesIndexString(IdInstance) - Method in class main.ml2db.mulan.mulanarff.IdMultiLabelInstances


getAttributesNames() - Method in class main.ml2db.mulan.attributesfiltered.AttFiltArff


getAttributesNamesList() - Method in class main.ml2db.weka.AttributeFactory


getAttributesNamesString(IdInstance) - Method in class main.ml2db.mulan.mulanarff.IdMultiLabelInstances


getAttributesNameTypeMap() - Method in class main.ml2db.mulan.mulanarff.MulanAttributeFactory


getAttributesNameTypeMap() - Method in class main.ml2db.weka.AttributeFactory


getAttributesStringPool() - Method in class main.ml2db.mulan.mulanarff.IdMultiLabelInstances


getAttributesValues(String) - Method in class main.ml2db.weka.InstAttValueMap


getAverageUncertainty(double[]) - Static method in class main.ml2db.al.utility.confidence.ConfidenceCalculator


getBalance() - Method in class main.ml2db.al.utility.globaldistance.GlobalDistanceMetric


getBaseDataToBePredicted() - Method in class main.ml2db.mulan.predict.PredictionsSaver


getBaseDotAllInstances() - Method in class main.ml2db.graphviz.GraphVizArff


getBaseFileName() - Method in class main.ml2db.Serializer


getBinaryAttributeValuesSet() - Method in class main.ml2db.weka.AttributeFactory
:   Get the possible attribute values for a yes/no attribute.

getBinaryRelevanceWrappedClassifier(Classifier, String) - Static method in class main.ml2db.mulan.learn.MulanLearners


getBPMLLBackPropagation() - Static method in class main.ml2db.mulan.learn.MulanLearners


getBRk1NN() - Static method in class main.ml2db.mulan.learn.MulanLearners
:   (Binary Relevance Nearest Neighbours)

getBRk1NNAL() - Static method in class main.ml2db.mulan.learn.MulanLearners
:   (Binary Relevance Nearest Neighbours)

getBRk1NNALJaccard() - Static method in class main.ml2db.mulan.learn.MulanLearners
:   (Binary Relevance Nearest Neighbours)

getBRk1NNALJaccardEuclidean() - Static method in class main.ml2db.mulan.learn.MulanLearners
:   Binary Relevance Nearest Neighbours with jaccard+euclidean distance

getBRk1NNManhattan() - Static method in class main.ml2db.mulan.learn.MulanLearners
:   Binary Relevance Nearest Neighbours learner (Active Learning version)
    with Manhattan distance instead of default Euclidean distance.

getBRk2NNManhattan() - Static method in class main.ml2db.mulan.learn.MulanLearners


getBRk5NNAL() - Static method in class main.ml2db.mulan.learn.MulanLearners
:   (Binary Relevance Nearest Neighbours)

getBRkNN(int) - Static method in class main.ml2db.mulan.learn.MulanLearners
:   public static MulanLearner getBRk1NNAL() { return
    MulanLearner.getBRkNNAL(MulanLearners.K1); }

getBRkNNAL(int) - Static method in class main.ml2db.mulan.learn.MulanLearners


getByAttributesString(String) - Method in class main.ml2db.mulan.mulanarff.IdMultiLabelInstances
:   Finds an instance having all these attributes names as present (=1)
    attributes.

getBySparseInstanceString(String) - Method in class main.ml2db.mulan.mulanarff.IdMultiLabelInstances
:   Find instances having the same sparse instance string ( indexed
    attributes string {0 1, 3 1, 5 1} including all feature and class
    attributes).

getCardinalityDistribution() - Method in class main.ml2db.mulan.mulanarff.Statistics


getClassAttribute() - Method in class main.ml2db.weka.AttributeFactory
:   Get last attribute as class attribute

getClassAttributes() - Method in class main.ml2db.mulan.mulanarff.IdMultiLabelInstances


getClassAttributes() - Method in class main.ml2db.mulan.mulanarff.MulanAttributeFactory
:   Get the Mulan class attributes

getClassName() - Method in class main.ml2db.weka.DataSetGenerator


getClassName() - Method in class main.ml2db.weka.DataSetManager


getColumnDefinitions() - Static method in class main.ml2db.al.database.AlEvaluationTable


getColumnDefinitions() - Static method in class main.ml2db.al.database.AlExperimentsTable


getColumnDefinitions() - Static method in class main.ml2db.al.database.QueryTable


getColumnDefinitions() - Static method in class main.ml2db.al.database.UtilityTable


getColumnDefinitions() - Static method in class main.ml2db.mulan.learn.crosseval.EvaluationResultsTable
:   Add timestamps and identifiers to the cross evaluation results

getColumnDefinitions() - Static method in class main.ml2db.mulan.predict.PredictionsExperimentTable


getColumnDefinitions() - Static method in class main.ml2db.mulan.predict.PredictionsRecordTable


getColumnDefinitions() - Static method in class main.ml2db.weka.ArffRecordsTable
:   Creates the table for the ARFF file records

getConfidenceCalculator() - Method in class main.ml2db.al.utility.confidence.ConfidenceMetric


getConfidenceCalculator() - Method in class main.ml2db.al.utility.hybrid.GlobalDistanceWithConfidenceMetric


getConfidenceCalculator() - Static method in class test.ml2db.al.utility.confidence.ConfidenceCalculatorTest


getConfidenceMetric() - Static method in class test.ml2db.al.utility.confidence.ConfidenceMetricTest


getConfidences(MultiLabelLearner, IdInstance) - Static method in class main.ml2db.al.utility.confidence.ConfidenceCalculator
:   // input: trained model // actions: get MultiLabelOutput from trained
    model using // .makePredictions(Instance), // get confidence[] --> one
    confidence for each class label

getCorrectClassAttributes() - Static method in class test.ml2db.mulan.attributesfilter.AttFiltArffTest


getCorrectFeatureAttributes() - Static method in class test.ml2db.mulan.attributesfilter.AttFiltArffTest


getCurrentNeighbourhoodName() - Method in class main.ml2db.al.utility.pickers.byfrequency.NearestNeighboursByFreqUtility


getDataArffProperties() - Method in class main.ml2db.weka.Arff


getDataDb() - Method in class main.ml2db.DbManagers


getDataDbManager() - Method in class main.ml2db.weka.Arff


getDataLoader() - Method in class main.ml2db.mulan.mulanarff.MulanDataSetManager


getDataLoader() - Method in class main.ml2db.weka.DataSetManager


getDataProperties() - Method in class main.ml2db.weka.DataSetDbLoader


getDataPropsFilePath() - Method in class main.ml2db.weka.Arff


getDataSaver() - Method in class main.ml2db.al.ActiveLearning


getDataSetChecker() - Method in class main.ml2db.weka.DataSetManager


getDataSetCheckerKO() - Static method in class test.ml2db.weka.DataSetCheckerTest


getDataSetCheckerOK() - Static method in class test.ml2db.weka.DataSetCheckerTest


getDataSetGenerator() - Method in class main.ml2db.mulan.mulanarff.MulanAttributeFactory


getDataSetGenerator() - Method in class main.ml2db.mulan.mulanarff.MulanDataSetManager


getDataSetGenerator() - Method in class main.ml2db.mulan.mulanarff.MulanInstancesFiller


getDataSetGenerator() - Method in class main.ml2db.weka.AttributeFactory


getDataSetGenerator() - Method in class main.ml2db.weka.DataSetManager


getDataSetGenerator() - Method in class main.ml2db.weka.InstancesFiller


getDataSetGenerator() - Static method in class test.ml2db.weka.DataSetGeneratorTest


getDataSetGeneratorTrainFile() - Static method in class test.ml2db.mulan.mulanarff.MulanDataSetGeneratorTest
:   public static MulanDataSetGenerator getDataSetGeneratorArff1() { return
    MulanDataSetManagerTest.getDataSetManagerArff1().getDataSetGenerator(); }

getDataSetManager() - Method in class main.ml2db.mulan.mulanarff.MulanArff


getDataSetManager() - Method in class main.ml2db.weka.Arff


getDataSetManager() - Method in class main.ml2db.weka.DataSetDbLoader


getDataSetManager() - Method in class main.ml2db.weka.DataSetWriter


getDataSetManager() - Static method in class test.ml2db.weka.DataSetManagerTest


getDataSetManagerArff1() - Static method in class test.ml2db.mulan.mulanarff.MulanDataSetManagerTest


getDataSetManagerTrainFile() - Static method in class test.ml2db.mulan.mulanarff.MulanDataSetManagerTest


getDataSetName() - Method in class main.ml2db.weka.DataSetManager


getDataSetWithNoXmlProperty() - Static method in class test.ml2db.weka.DataSetManagerTest


getDataSetWriter() - Method in class main.ml2db.weka.DataSetManager


getDataSetWriter() - Static method in class test.ml2db.weka.DataSetWriterTest


getDbConn() - Method in class main.ml2db.weka.Arff


getDbConn() - Method in class main.ml2db.weka.ArffProps


getDbConn() - Method in class main.ml2db.weka.ImportArff


getDbLoader() - Static method in class test.ml2db.mulan.mulanarff.MulanDataSetDbLoaderTest


getDbLoader() - Static method in class test.ml2db.weka.DataSetDbLoaderTest


getDbMan() - Method in class main.ml2db.mulan.mulanarff.IdMultiLabelInstances


getDbMan() - Method in class main.ml2db.mulan.predict.PredictionsSaver


getDbManager() - Static method in class test.ml2db.dataharness.DatabaseTest


getDbManagers() - Method in class main.ml2db.mulan.attributesfiltered.AttFilter


getDbManagers() - Method in class main.ml2db.mulan.learn.crosseval.CrossInputManager


getDbManagers() - Method in class main.ml2db.mulan.mulanarff.database.MulanArffRecord


getDbManagers() - Method in class main.ml2db.weka.Arff


getDbManagers() - Static method in class test.ml2db.dataharness.TestProjectParameters


getDbManagers() - Static method in class test.ml2db.weka.ArffPropsTableTest


getDescription() - Method in class main.ml2db.weka.ImportArff


getDesired() - Method in class main.ml2db.mulan.mulanarff.NeighboursPrinter


getDirectedGraphvizString(IdInstance) - Static method in class main.ml2db.graphviz.GraphvizInstance


getDirectoryForSerializedObject() - Method in class main.ml2db.Serializer


getDistance(Instance, Instance) - Method in class main.ml2db.al.utility.globaldistance.DistancesCalculator


getDistanceCalculator() - Method in class main.ml2db.al.utility.globaldistance.GlobalDistanceMetric


getDistanceFunction() - Method in class main.ml2db.al.learning.ExperimentDetails


getDistanceFunction() - Method in class main.ml2db.al.utility.globaldistance.DistancesCalculator


getDistances() - Method in class main.ml2db.al.utility.globaldistance.DistancesStorage


getDistances(Instance, int) - Method in class main.ml2db.al.utility.globaldistance.GlobalDistanceMetric


getDistances(String, int) - Method in class main.ml2db.al.utility.globaldistance.GlobalPositionStore
:   Find the distance of an instance to all labelled instances.

getDistancesCalculatorOnTestSet() - Static method in class test.ml2db.al.utility.globaldistance.DistancesCalculatorTest


getDistancesFileName() - Method in class main.ml2db.al.utility.globaldistance.DistancesCalculator


getDistancesStorageInt(int) - Static method in class test.ml2db.al.utility.globaldistance.DistancesStorageTest


getDotSource() - Method in class main.ml2db.graphviz.GraphViz
:   Returns the graph's source description in dot language.

getEndTime() - Method in class main.ml2db.mulan.learn.evaluation.EvaluatorNoAUC


getEnsembleOfClassifierChains(int) - Static method in class main.ml2db.mulan.learn.MulanLearners
:   The Classifier Chain model ( CC ) involves | L | binary classifiers as in
    BM .

getEuclideanDistance() - Method in class main.ml2db.weka.JaccardEuclideanDistance


getEvaluation() - Static method in class test.ml2db.mulan.learn.database.ResultsFormatterTest


getEvaluationMeasures() - Method in class main.ml2db.mulan.learn.crosseval.CrossOutputManager


getEvaluationResultsFile() - Method in class main.ml2db.al.learning.ExperimentDetails


getEvaluationResultsRows() - Method in class main.ml2db.al.ActiveLearning


getEvaluationResultsTable() - Method in class main.ml2db.mulan.learn.crosseval.CrossOutputManager


getEvaluationResultsTable() - Static method in class test.ml2db.mulan.learn.EvaluationResultsTableTest


getEvaluationRow(TrainTestEvaluator, boolean, boolean) - Static method in class test.ml2db.mulan.learn.traintest.TrainTestEvaluatorTest


getEvaluator() - Method in class main.ml2db.mulan.learn.traintest.TrainTestExperimenter


getExamplesPerLabel() - Method in class main.ml2db.mulan.mulanarff.Statistics


getExpectedArff() - Static method in class test.ml2db.weka.DataSetManagerTest


getExpectedMulanTestFile() - Static method in class test.ml2db.mulan.mulanarff.MulanArffTest


getExpectedMulanTrainFile() - Static method in class test.ml2db.mulan.mulanarff.MulanArffTest


getExpectedMulanXml2() - Static method in class test.ml2db.mulan.mulanarff.MulanArffTest


getExpectedMulanXmlTestFile() - Static method in class test.ml2db.mulan.mulanarff.MulanArffTest


getExpectedMulanXmlTrainFile() - Static method in class test.ml2db.mulan.mulanarff.MulanArffTest


getExperiment() - Method in class main.ml2db.mulan.learn.crosseval.CrossInputManager


getExperimentDbManager() - Method in class main.ml2db.mulan.learn.crosseval.CrossOutputManager
:   public MulanCrossEvaluator getEvaluator() { return m\_evaluator; }

getExperimentDetails() - Method in class main.ml2db.al.ActiveLearning


getExperimenter() - Method in class main.ml2db.al.learning.AlOutputManager


getExperimenter() - Method in class main.ml2db.mulan.learn.crosseval.CrossOutputManager


getExperimenter() - Method in class main.ml2db.mulan.learn.evaluation.EvaluatorNoAUC


getExperimenter() - Method in class main.ml2db.mulan.learn.traintest.TrainTestEvaluator


getExperimenter() - Method in class main.ml2db.mulan.learn.traintest.TrainTestOutputManager


getExperimentId() - Method in class main.ml2db.al.ActiveLearning


getExperimentId() - Method in class main.ml2db.al.learning.ExperimentDetails


getExperimentManagerTestset() - Static method in class test.ml2db.al.utility.neighbourhood.NeighbourhoodExperimentDetailsTest


getExperimentManagerTestsetWithPositioning() - Static method in class test.ml2db.al.utility.globaldistance.DistanceExperimentDetailsTest


getExperimentManagerTrainset() - Static method in class test.ml2db.al.utility.neighbourhood.NeighbourhoodExperimentDetailsTest


getExperimentManagerTrainsetWithPositioning() - Static method in class test.ml2db.al.utility.globaldistance.DistanceExperimentDetailsTest


getExperimentTable() - Method in class main.ml2db.mulan.predict.PredictionsSaver


getExperimentTableRowSaver() - Method in class main.ml2db.al.database.AlDataSaver


getExpManagerTestsetWithRandomAttPicker() - Static method in class test.ml2db.al.utility.pickers.RandomAttributePickerTest


getExpManOnTestsetWithConfidenceMetric() - Static method in class test.ml2db.al.utility.confidence.ConfidenceMetricTest


getExpManTestsetWithGlobalPositAndConfidence() - Static method in class test.ml2db.al.utility.globaldistance.DistanceExperimentDetailsTest


getFalseNegatives() - Method in class main.ml2db.mulan.learn.evaluation.MicroFMeasurePrinter


getFalsePositives() - Method in class main.ml2db.mulan.learn.evaluation.MicroFMeasurePrinter


getFeatureAttributes() - Method in class main.ml2db.weka.JaccardDistanceStored


getFeaturesByDescendentFrequency() - Method in class main.ml2db.al.utility.pickers.byfrequency.ByFrequencyPicker


getFeatureToInstancesMap() - Method in class main.ml2db.al.utility.pickers.RandomInstancePicker


getFile() - Method in class main.ml2db.mulan.classifier.lazy.BRkNNVisible


getFileForSerializedObject() - Method in class main.ml2db.Serializer


getFileName() - Method in class main.ml2db.al.database.AlDataSaver


getFilePath() - Method in class main.ml2db.al.database.AlDataSaver
:   File to save the experiment data to (as backup)

getFilePath() - Method in class main.ml2db.al.utility.globaldistance.DistancesCalculator


getFilePath() - Method in class main.ml2db.mulan.predict.PredictionsSaver


getFilePath() - Method in class main.ml2db.weka.DataSetWriter


getFileRole(String) - Static method in class test.ml2db.mulan.attributesfilter.AttFiltArffTest
:   Returns the role ("train" or "test") of the file for a potential
    train/test evaluation.

getFilteredArffName(int, int, int) - Static method in class main.ml2db.mulan.attributesfiltered.AttFiltArff


getFilteredArffs(int, int) - Static method in class test.ml2db.mulan.learn.traintest.EvaluationMetricsTest
:   public static void basicPredictionsTest(TrainTestExperimenter exp, String
    expectedPredictions) {
    // emit predictions int train =
    exp.getInputManager().getTrainArffRecord().getArffId(); int test =
    exp.getInputManager().getTestArffRecord().getArffId(); String
    machLearningDb = exp.getOutputManager().getExperimentDbManager()
    .getDatabaseName();
    System.out.println("Now predicting from:"); System.out.println("train " +
    train + ", test " + test + ", results written on db: " + machLearningDb);
    String predictionsFilePath = PredictionsManager.run(train, test,
    TestProjectParameters.getDbManagers(),
    TestProjectParameters.TEST\_PREDICTIONS\_FOLDER,
    MulanLearners.getBRk1NNAL(), false);
    // read predictions from file System.out.println("Predictions at: " +
    predictionsFilePath + "\n\n");
    assertEquals(expectedPredictions,
    FileUtils.getStringFromFile(predictionsFilePath)); }

getFilterManager() - Method in class main.ml2db.mulan.attributesfiltered.AttFiltArff


getFirstInstance() - Method in class main.ml2db.mulan.mulanarff.IdMultiLabelInstances


getFirstTestArffFromDb() - Static method in class test.ml2db.dataharness.LoadFirstValidArff


getFirstTestArffFromDb() - Static method in class test.ml2db.mulan.mulanarff.MulanArffTest
:   public static int getFirstFilteredTestArff() { ArffPropsTableReader
    reader = ArffPropsTableReaderTest.getReader1(); // return the first arff
    having different identifiers as "old test id" // and
    "id the attributes were taken from (train set)"
    // return the first arff id containing "test" in its pathname return
    reader.getIdByArffPath("\_with\_attr\_of\_"); }
    public static int getFirstFilteredTrainArff() { ArffPropsTableReader
    reader = ArffPropsTableReaderTest.getReader1(); // return the first arff
    having different identifiers as "old test id" // and
    "id the attributes were taken from (train set)"
    // return the first arff id containing "test" in its pathname return
    reader.getIdByArffPath("\_with\_attr\_of\_"); }

getFirstTestArffInstancesFromDb() - Static method in class test.ml2db.dataharness.LoadFirstValidArff


getFirstTrainArffFromDb() - Static method in class test.ml2db.dataharness.LoadFirstValidArff


getFirstTrainArffFromDb() - Static method in class test.ml2db.mulan.mulanarff.MulanArffTest


getFirstTrainArffInstancesFromDb() - Static method in class test.ml2db.dataharness.LoadFirstValidArff


getFirstTrainPathFromDb() - Static method in class test.ml2db.dataharness.LoadFirstValidArff
:   Get the arff path and xml path

getFirstValidArff2FromDb() - Static method in class test.ml2db.mulan.mulanarff.MulanArffTest


getFrequencyToFeatureMap() - Method in class main.ml2db.al.utility.pickers.byfrequency.ByFrequencyPicker


getFullTrainingSet() - Method in class main.ml2db.mulan.predict.PredictionsSaver


getGlobPositionStore(int) - Method in class main.ml2db.al.utility.globaldistance.GlobalPositionStore


getGraphImage(String, String) - Method in class main.ml2db.graphviz.GraphViz
:   Returns the graph as an image in binary format.

getGraphViz() - Static method in class test.ml2db.graphviz.GraphVizTest
:   Test method for `GraphViz.GraphViz()`.

getGraphVizArffColouredTest() - Static method in class test.ml2db.graphviz.GraphVizArffColouredTest


getGraphVizArffColouredTrain() - Static method in class test.ml2db.graphviz.GraphVizArffColouredTest


getGraphVizArffTest() - Static method in class test.ml2db.graphviz.GraphVizArffTest


getGraphVizArffTrain() - Static method in class test.ml2db.graphviz.GraphVizArffTest


getHeaderString(String) - Static method in class main.ml2db.weka.Arff
:   Returns the header part of the arff representation (everything before the
    \@data label)

getHierarchicalMultilabel(MultiLabelLearnerBase, String) - Static method in class main.ml2db.mulan.learn.MulanLearners


getHomer() - Static method in class main.ml2db.mulan.learn.MulanLearners


getId2IdInstanceMap() - Method in class main.ml2db.mulan.mulanarff.IdMultiLabelInstances


getId2WekaInstanceMap() - Method in class main.ml2db.mulan.mulanarff.IdMultiLabelInstances


getIdByArffPath(String) - Method in class main.ml2db.weka.ArffRecordsTable
:   Returns the first arff id in the table whose arff path matches the given
    string (or part of it)

getIdByArffPath(String, String, DbManagers) - Method in class main.ml2db.weka.ArffRecordsTable
:   Returns the first arff id in the table whose arff path matches the given
    string (or part of it).

getIdInstance(Instance) - Method in class main.ml2db.mulan.mulanarff.IdMultiLabelInstances


getIdInstance(String) - Method in class main.ml2db.mulan.mulanarff.IdMultiLabelInstances


getIdInstances() - Method in class main.ml2db.weka.JaccardDistanceStored


getIdInstancesByAttributesString(String) - Method in class main.ml2db.mulan.mulanarff.IdMultiLabelInstances


getIdMultiLabelInstancesTestset() - Static method in class test.ml2db.mulan.mulanarff.IdMultiLabelInstancesTest


getIdMultiLabelInstancesTrainset() - Static method in class test.ml2db.mulan.mulanarff.IdMultiLabelInstancesTest


getImagesPath() - Method in class main.ml2db.al.learning.ExperimentDetails


getIndexOfIdInstance(String) - Method in class main.ml2db.mulan.mulanarff.IdMultiLabelInstances


getInformationPerNeighbour() - Method in class main.ml2db.al.utility.neighbours.Neighbourhood
:   Returns the fraction of information that each neighbour contributes to
    this instance (1 / number of neighbours )

getInputAndOutputDbManagersDbManager() - Static method in class test.ml2db.InputAndOutputDbManagersTest


getInputDbReader() - Method in class main.ml2db.weka.Arff


getInputManager() - Method in class main.ml2db.mulan.learn.crosseval.CrossExperimenter


getInputManager() - Method in class main.ml2db.mulan.learn.traintest.TrainTestExperimenter


getInsertDataSqlStatement() - Method in class test.ml2db.dataharness.DataTableFourTest


getInsertDataSqlStatement() - Method in class test.ml2db.dataharness.DataTableOneTest


getInsertDataSqlStatement() - Method in class test.ml2db.dataharness.DataTableTest


getInsertDataSqlStatement() - Method in class test.ml2db.dataharness.DataTableThreeTest


getInsertDataSqlStatement() - Method in class test.ml2db.dataharness.DataTableTwoTest


getInst2LabelsPartitionMap() - Method in class main.ml2db.mulan.mulanarff.IdMultiLabelInstances


getInst2PartitionMapString(TreeMap<IdInstance, boolean[]>) - Static method in class main.ml2db.mulan.mulanarff.IdMultiLabelInstances


getInstance(MulanInstancesFiller) - Static method in class test.ml2db.mulan.mulanarff.MulanInstancesFillerTest


getInstance1\_2\_empty\_ArffId() - Static method in class test.ml2db.mulan.learn.traintest.EvaluationMetricsTest


getInstance1\_2ArffId() - Static method in class test.ml2db.mulan.learn.traintest.EvaluationMetricsTest


getInstance2NonZeroFeatAttIndexes() - Method in class main.ml2db.weka.JaccardDistanceStored


getInstance3ArffId() - Static method in class test.ml2db.mulan.learn.traintest.EvaluationMetricsTest


getInstanceAndNeighboursMap() - Method in class main.ml2db.mulan.mulanarff.NeighboursPrinter


getInstanceAttributeData() - Method in class main.ml2db.weka.DataSetDbLoader


getInstanceAttributeMap() - Method in class main.ml2db.weka.DataSetGenerator


getInstanceAttributeMap() - Static method in class test.ml2db.dataharness.DataTwo


getInstanceClassData() - Method in class main.ml2db.weka.DataSetDbLoader


getInstanceClassMap() - Method in class main.ml2db.weka.DataSetGenerator


getInstanceClassMap() - Static method in class test.ml2db.dataharness.DataTwo


getInstanceNamesStringPool() - Method in class main.ml2db.mulan.mulanarff.IdMultiLabelInstances


getInstances() - Method in class main.ml2db.al.utility.globaldistance.DistancesCalculator


getInstances() - Method in class main.ml2db.mulan.measuremetrics.AnnotationsReader


getInstances() - Method in class main.ml2db.mulan.mulanarff.MulanArff


getInstances() - Method in class main.ml2db.mulan.mulanarff.NeighboursPrinter


getInstances() - Method in class main.ml2db.weka.Arff


getInstances() - Method in class main.ml2db.weka.DataSetGenerator


getInstances() - Method in class main.ml2db.weka.JaccardDistanceStored


getInstancesFactory() - Method in class main.ml2db.weka.DataSetGenerator


getInstancesNames() - Method in class main.ml2db.al.ActiveLearning


getInstancesNames() - Method in class main.ml2db.mulan.mulanarff.MulanArff


getInstancesNames() - Method in class main.ml2db.weka.DataSetGenerator


getInstancesNamesFromFile() - Method in class main.ml2db.mulan.mulanarff.database.MulanArffRecord
:   Gets the instances names directly from the comment line above each
    instance

getInstancesNamesFromFile(String) - Static method in class main.ml2db.mulan.mulanarff.database.MulanArffRecord
:   Gets the instances names directly from the comment line above each
    instance in the ARFF file (eg.

getInstancesOfTrainedLerner1() - Static method in class test.ml2db.al.utility.confidence.ConfidenceCalculatorTest


getInstancesOfTrainedLerner2() - Static method in class test.ml2db.al.utility.confidence.ConfidenceCalculatorTest


getInstancesString(String) - Static method in class main.ml2db.weka.Arff
:   Returns the instances part of the arff representation (everything after
    the \@data label)

getInstancesTree() - Method in class main.ml2db.al.utility.neighbours.NeighbourhoodNetwork


getInstanceStringPool() - Method in class main.ml2db.mulan.mulanarff.IdMultiLabelInstances


getInstanceToFeaturesMap() - Method in class main.ml2db.al.utility.pickers.LabelRandomPicker


getInstanceToFeaturesMap() - Method in class main.ml2db.al.utility.pickers.RandomAttributePicker


getInstanceToFeaturesMap() - Method in class main.ml2db.al.utility.pickers.RandomAttributeSetPicker


getInstanceToFeaturesMap() - Method in class main.ml2db.al.utility.pickers.RandomInstancePicker


getInstanceToFeaturesMap() - Method in class main.ml2db.al.utility.pickers.RandomLabelsPicker


getInstanceToFeaturesMap() - Method in class main.ml2db.al.utility.pickers.RandomNeigbourhoodPicker


getInstanceToFeaturesMap() - Method in class main.ml2db.al.utility.pickers.RandomPicker


getInstAttValueMapNominal() - Static method in class test.ml2db.weka.InstAttValueMapTest


getInstAttValueMapNumeric() - Static method in class test.ml2db.weka.InstAttValueMapTest


getInstNames2AttrIndexStringMap() - Method in class main.ml2db.mulan.mulanarff.IdMultiLabelInstances


getInstNames2AttrNamesStringMap() - Method in class main.ml2db.mulan.mulanarff.IdMultiLabelInstances


getInstSparseStringToLabelsString() - Method in class main.ml2db.mulan.mulanarff.IdMultiLabelInstances


getIntersection(TreeSet<Integer>, TreeSet<Integer>) - Method in class main.ml2db.weka.JaccardDistance
:   Calculate the size of the intersection of set A (feature attributes of
    instance A) and set B (feature attributes of instance B), that is, the
    non-zero attributes they have in common.

getIntersection(TreeSet<Integer>, TreeSet<Integer>) - Method in class main.ml2db.weka.JaccardDistanceStored
:   Calculate the size of the intersection of set A (feature attributes of
    instance A) and set B (feature attributes of instance B), that is, the
    non-zero attributes they have in common.

getInvertSelection() - Method in class main.ml2db.weka.JaccardDistanceStored


getIsNeededBy() - Method in class main.ml2db.al.utility.neighbours.Neighbourhood


getJaccardDistanceStore() - Method in class main.ml2db.weka.JaccardDistanceStored


getJaccardDistanceTestSet() - Static method in class main.ml2db.weka.JaccardDistanceTest


getLabelCardinality() - Method in class main.ml2db.mulan.mulanarff.Statistics


getLabelDensity() - Method in class main.ml2db.mulan.mulanarff.Statistics


getLabelIndices() - Method in class main.ml2db.al.utility.neighbours.LinearMultiLabelNNSearch


getLabelled() - Method in class main.ml2db.al.ActiveLearning


getLabelled() - Method in class main.ml2db.al.utility.globaldistance.GlobalDistanceMetric


getLabelledArffId() - Method in class main.ml2db.al.learning.ExperimentDetails


getLabelledDistances(Instance) - Method in class main.ml2db.al.utility.globaldistance.GlobalDistanceMetric


getLabelledInstances() - Method in class main.ml2db.al.learning.ExperimentDetails


getLabelPowersetJ48C45() - Static method in class main.ml2db.mulan.learn.MulanLearners
:   From Wikipedia: C4.5 is an algorithm used to generate a decision tree
    developed by Ross Quinlan.

getLabelPowersetSMO() - Static method in class main.ml2db.mulan.learn.MulanLearners


getLabelPowersetSMOReg() - Static method in class main.ml2db.mulan.learn.MulanLearners


getLabelPowersetWrappedClassifier(Classifier, String) - Static method in class main.ml2db.mulan.learn.MulanLearners


getLabels() - Method in class main.ml2db.mulan.measuremetrics.AnnotationsReader


getLabels2PartitionMap() - Method in class main.ml2db.mulan.mulanarff.IdMultiLabelInstances


getLabelsArray() - Method in class main.ml2db.mulan.measuremetrics.AnnotationsReader


getLabelsets() - Method in class main.ml2db.mulan.mulanarff.Statistics


getLabelsString(IdInstance) - Method in class main.ml2db.mulan.mulanarff.IdMultiLabelInstances
:   Get a separated list of the labels assigned to this instance.

getLabInstances() - Method in class main.ml2db.al.ActiveLearning


getLabInstances() - Method in class main.ml2db.al.learning.ExperimentDetails


getLearner() - Method in class main.ml2db.mulan.learn.crosseval.CrossExperimenter


getLearner() - Method in class main.ml2db.mulan.learn.MulanLearner


getLearner() - Method in class main.ml2db.mulan.learn.MulanSerializer


getLearnerBaseType() - Method in class main.ml2db.mulan.learn.MulanLearner


getLearnerFullName() - Method in class main.ml2db.mulan.learn.MulanLearner
:   Get the full type of a learner: the name of the multi-label learner
    base/wrapper plus the weka or mulan algorithm/classifier name

getLearners() - Static method in class main.ml2db.mulan.learn.MulanLearners


getLearnerType() - Method in class main.ml2db.mulan.learn.MulanLearner


getLinearNNSearch() - Method in class main.ml2db.al.utility.pickers.byfrequency.NearestNeighboursByFreqUtility


getLoadedMulanArff(String, MulanArff) - Static method in class test.ml2db.mulan.mulanarff.MulanArffTest


getLocalDbManagers() - Static method in class main.ml2db.ProjectParameters


getLog() - Method in class main.ml2db.weka.DataSetWriter


getLogFile() - Method in class main.ml2db.al.ActiveLearning


getLogFile() - Method in class main.ml2db.mulan.mulanarff.IdMultiLabelInstances


getLogFile() - Method in class main.ml2db.Serializer


getLogFilePath() - Method in class main.ml2db.mulan.predict.PredictionsSaver


getManager() - Method in class main.ml2db.weka.DataSetChecker


getManager() - Method in class main.ml2db.weka.DataSetGenerator


getMap() - Method in class main.ml2db.al.utility.confidence.ConfidencesStorage


getMap(String, String) - Method in class main.ml2db.weka.DataSetDbLoader


getMeasureFieldNames() - Static method in class main.ml2db.mulan.learn.crosseval.EvaluationResultsTable
:   A hashtable containing for each evaluation measure the corresponding
    table field name.

getMeasureFields() - Static method in class main.ml2db.mulan.learn.crosseval.EvaluationResultsTable
:   A hashtable containing for each measure field name the corresponding
    table field object (name + sql data type).

getMeasureNames() - Static method in class main.ml2db.mulan.learn.evaluation.EvaluationParameters
:   Get a list containing the evaluation measure names.

getMeasureTableField(String) - Static method in class main.ml2db.mulan.learn.crosseval.EvaluationResultsTable
:   For each evaluation measure, gets the corresponding table field The field
    name is the evaluation measure name, all lower-case, with spaces and
    dashes substituted with underscores.

getMetricName() - Method in class main.ml2db.al.utility.confidence.ConfidenceMetric


getMetricName() - Method in class main.ml2db.al.utility.globaldistance.GlobalDistanceMetric


getMetricName() - Method in class main.ml2db.al.utility.hybrid.GlobalDistanceWithConfidenceMetric


getMetricName() - Method in class main.ml2db.al.utility.neighbours.NeighbourhoodNetwork


getMetricName() - Method in class main.ml2db.al.utility.pickers.byfrequency.AttributesByFrequencyPicker


getMetricName() - Method in class main.ml2db.al.utility.pickers.byfrequency.AttSetByFrequencyPicker


getMetricName() - Method in class main.ml2db.al.utility.pickers.byfrequency.ByFrequencyPicker


getMetricName() - Method in class main.ml2db.al.utility.pickers.byfrequency.LabelsByFreqPicker


getMetricName() - Method in class main.ml2db.al.utility.pickers.byfrequency.NearestNeighboursByFreqUtility


getMetricName() - Method in class main.ml2db.al.utility.pickers.LabelRandomPicker


getMetricName() - Method in class main.ml2db.al.utility.pickers.RandomAttributePicker


getMetricName() - Method in class main.ml2db.al.utility.pickers.RandomAttributeSetPicker


getMetricName() - Method in class main.ml2db.al.utility.pickers.RandomInstancePicker


getMetricName() - Method in class main.ml2db.al.utility.pickers.RandomLabelsPicker


getMetricName() - Method in class main.ml2db.al.utility.pickers.RandomNeigbourhoodPicker


getMetricName() - Method in class main.ml2db.al.utility.pickers.RandomPicker


getMetricName() - Method in class main.ml2db.al.utility.UtilityMetric


getMlResultsDb() - Method in class main.ml2db.DbManagers


getMulanArff(String) - Static method in class test.ml2db.mulan.mulanarff.MulanArffTest


getMulanArff1() - Static method in class test.ml2db.mulan.mulanarff.MulanArffTest


getMulanArff1LoadedAndWritten() - Static method in class test.ml2db.mulan.mulanarff.MulanArffTest


getMulanArffNumericAttributes() - Static method in class test.ml2db.mulan.mulanarff.MulanArffTest


getMulanArffOldTestset() - Static method in class test.ml2db.mulan.mulanarff.MulanArffTest


getMulanArffTestsetLoadedAndWritten() - Static method in class test.ml2db.mulan.mulanarff.MulanArffTest


getMulanArffTrainLoadedAndWritten() - Static method in class test.ml2db.mulan.mulanarff.MulanArffTest


getMulanArffTrainset() - Static method in class test.ml2db.mulan.mulanarff.MulanArffTest


getMulanEvaluator2() - Static method in class test.ml2db.mulan.learn.CrossEvaluatorTest


getMulanEvaluatorTestSet() - Static method in class test.ml2db.mulan.learn.CrossEvaluatorTest


getMulanExperimenter(int) - Static method in class test.ml2db.mulan.learn.ExperimenterTest


getMulanExperimenter(int, String, String) - Static method in class test.ml2db.mulan.learn.ExperimenterTest


getMulanExperimenter2() - Static method in class test.ml2db.mulan.learn.ExperimenterTest


getMulanExperimenterFilterTrainset() - Static method in class test.ml2db.mulan.learn.ExperimenterTest


getMulanLearnerOneR() - Static method in class test.ml2db.mulan.learn.MulanLearnerTest


getMulanLearnerZeroR() - Static method in class test.ml2db.mulan.learn.MulanLearnerTest


getMulanSerializer2() - Static method in class test.ml2db.mulan.mulanarff.SerializerTest


getMulanSerializerFilterTrainset() - Static method in class test.ml2db.mulan.mulanarff.SerializerTest


getMulanTrainTestEvaluatorBRk1NN() - Static method in class test.ml2db.mulan.learn.traintest.TrainTestEvaluatorTest


getMulanTrainTestExperBRk1NN() - Static method in class test.ml2db.mulan.learn.traintest.TrainTestExperimenterTest


getMulanTrainTestExperBRk1NNById(int, int) - Static method in class test.ml2db.mulan.learn.traintest.TrainTestExperimenterTest


getMulanTrainTestExperById(int, int, MulanLearner) - Static method in class test.ml2db.mulan.learn.traintest.TrainTestExperimenterTest


getNaiveBayesBinaryRelevance() - Static method in class main.ml2db.mulan.learn.MulanLearners


getNaiveBayesLabelPowerset() - Static method in class main.ml2db.mulan.learn.MulanLearners


getName() - Method in class main.ml2db.mulan.learn.evaluation.FalseNegatives


getName() - Method in class main.ml2db.mulan.learn.evaluation.FalsePositives


getName() - Method in class main.ml2db.mulan.learn.evaluation.TruePositives


getName(Classifier) - Static method in class main.ml2db.mulan.learn.MulanLearner


getName(MultiLabelLearner) - Static method in class main.ml2db.mulan.learn.MulanLearner


getName(String) - Static method in class main.ml2db.mulan.learn.MulanLearner
:   remove the object number from the class name

getNameAttributeMap() - Method in class main.ml2db.weka.AttributeFactory


getNeededNeighbours() - Method in class main.ml2db.al.utility.neighbours.Neighbourhood


getNeededNetworkTestset() - Static method in class test.ml2db.al.utility.neighbourhood.NeighbourhoodNetworkTest


getNeededNetworkTrainset() - Static method in class test.ml2db.al.utility.neighbourhood.NeighbourhoodNetworkTest


getNeedsNominalOrNumeric() - Method in class main.ml2db.weka.InstAttValueMap


getNeighbours(Instance, IdMultiLabelInstances, int, boolean) - Method in class main.ml2db.al.utility.neighbours.LinearNNSearchSkipInstanceItself


getNeighboursSearcher() - Method in class main.ml2db.al.utility.neighbours.NeighbourhoodNetwork


getNeighboursSearcher() - Method in class main.ml2db.mulan.mulanarff.NeighboursPrinter


getNewEvaluator() - Method in class main.ml2db.mulan.learn.traintest.TrainTestExperimenter


getNewEvaluator() - Method in class main.ml2db.mulan.learn.traintest.TrainTestExperimenterSerialized


getNewFilteredArffs() - Static method in class test.ml2db.mulan.attributesfilter.AttFiltArffTest
:   Get the new, filtered, train and test arff files

getNewTestArffId() - Method in class main.ml2db.mulan.attributesfiltered.AttFilter


getNewTestDataSetGenerator() - Method in class test.ml2db.mulan.attributesfilter.AttFiltDataSetGeneratorTest


getNewTrainArffId() - Method in class main.ml2db.mulan.attributesfiltered.AttFilter


getNextStepQueryInstance(ActiveLearning, int) - Static method in class test.ml2db.al.ActiveLearningTest


getNNUtilityTestSet() - Static method in class test.ml2db.al.utility.neighbourhood.NearestNeighboursByFreqUtilityTest


getNNUtilityTrainSet() - Static method in class test.ml2db.al.utility.neighbourhood.NearestNeighboursByFreqUtilityTest


getNonZeroAttribIndexesOfInstance(Instance, Set<Attribute>) - Static method in class main.ml2db.mulan.mulanarff.IdMultiLabelInstances
:   Only input non-class (feature) attributes if the feature attributes
    indexes are desired.

getNonZeroAttributeNamesOfAnInstance(Instance, Set<Attribute>) - Static method in class main.ml2db.mulan.mulanarff.IdMultiLabelInstances
:   Get the non-zero valued attributes of an instance

getNonZeroLabelsOfAnInstance(Instance) - Method in class main.ml2db.mulan.mulanarff.IdMultiLabelInstances
:   Get the names of the non-zero valued class labels of an instance

getNonZeroLabelsOfTheseInstances() - Method in class main.ml2db.mulan.mulanarff.IdMultiLabelInstances
:   Get the names of the non-zero valued class labels of these instances

getNumAttributesWithExamples() - Method in class main.ml2db.mulan.mulanarff.Statistics


getNumberOfAttributes() - Method in class main.ml2db.mulan.mulanarff.MulanDataSetGenerator
:   Get the number of distinct feature attributes (all attribute values -
    class values)

getNumberOfAttributes() - Method in class main.ml2db.weka.DataSetGenerator


getNumberOfAttributes() - Method in class main.ml2db.weka.InstAttValueMap
:   Get how many attributes have a value (any value) for any instance in the
    map

getNumberOfClassValues() - Method in class main.ml2db.weka.DataSetGenerator


getNumberOfCoreProperties() - Method in class main.ml2db.weka.ArffProps


getNumberOfCoreProperties() - Method in class main.ml2db.weka.ArffPropsFromFile
:   The core ARFF properties, essential to generate a Weka ARFF file

getNumberOfInstanceAttributeCouples() - Method in class main.ml2db.mulan.mulanarff.MulanDataSetGenerator
:   Get the number of distinct instance-attribute couples of values by
    subtracting the number of instance-class\_attribute values from the total
    number.

getNumberOfInstanceAttributeCouples() - Method in class main.ml2db.weka.DataSetGenerator


getNumberOfInstanceAttributeCouples() - Method in class main.ml2db.weka.InstAttValueMap


getNumberOfInstanceClassCouples() - Method in class main.ml2db.weka.DataSetGenerator


getNumberOfInstances() - Method in class main.ml2db.mulan.learn.crosseval.Folds


getNumberOfInstances() - Method in class main.ml2db.mulan.measuremetrics.AnnotationsReader


getNumberOfInstances() - Method in class main.ml2db.weka.DataSetGenerator


getNumberOfInstancesFromDataMaps() - Method in class main.ml2db.weka.DataSetGenerator


getNumberOfInstancesWithClass() - Method in class main.ml2db.weka.DataSetGenerator


getNumberOfLabels() - Method in class main.ml2db.al.utility.neighbours.LinearMultiLabelNNSearch


getNumberOfLabels() - Method in class main.ml2db.mulan.learn.evaluation.MicroFMeasurePrinter


getNumberOfLabels() - Method in class main.ml2db.mulan.measuremetrics.AnnotationsReader


getNumberOfNearestNeighbours() - Method in class main.ml2db.al.utility.neighbours.Neighbourhood


getNumberOfNeedy() - Method in class main.ml2db.al.utility.neighbours.Neighbourhood


getNumberOfNeighbours() - Method in class main.ml2db.al.ActiveLearning


getNumberOfStatsProperties() - Method in class main.ml2db.weka.ArffProps


getNumberOfUnlabelledNeedy() - Method in class main.ml2db.al.utility.neighbours.Neighbourhood
:   Only consider instances that still need a label (exclude labelled
    instances).

getNumClassLabelsWithExamples() - Method in class main.ml2db.mulan.mulanarff.Statistics


getNumInstances() - Method in class main.ml2db.mulan.mulanarff.Statistics


getNumLabels() - Method in class main.ml2db.mulan.mulanarff.Statistics


getNumNominal() - Method in class main.ml2db.mulan.mulanarff.Statistics


getNumNumeric() - Method in class main.ml2db.mulan.mulanarff.Statistics


getNumPredictors() - Method in class main.ml2db.mulan.mulanarff.Statistics


getOldTestArffId() - Method in class main.ml2db.mulan.attributesfiltered.AttFilter


getOldTrainArffId() - Method in class main.ml2db.mulan.attributesfiltered.AttFilter


getOneRule() - Static method in class main.ml2db.mulan.learn.MulanLearners


getOptions() - Method in class main.ml2db.weka.JaccardDistanceStored


getOriginalSet() - Method in class main.ml2db.mulan.learn.crosseval.Folds


getOutputManager() - Method in class main.ml2db.al.learning.AlExperimenter


getOutputManager() - Method in class main.ml2db.mulan.learn.crosseval.CrossExperimenter


getOutputManager() - Method in class main.ml2db.mulan.learn.traintest.TrainTestExperimenter


getPercentPotentiallyLabelled() - Method in class main.ml2db.al.utility.neighbours.Neighbourhood


getPerceptron() - Static method in class main.ml2db.mulan.learn.MulanLearners


getPhi() - Method in class main.ml2db.mulan.mulanarff.Statistics


getPhiHistogram() - Method in class main.ml2db.mulan.mulanarff.Statistics
:   Calculates a histogram of phi correlations

getPositioningMetric() - Method in class main.ml2db.al.utility.globaldistance.GlobalPositionStore


getPositionsStore() - Method in class main.ml2db.al.utility.globaldistance.GlobalDistanceMetric


getPositionsStoreTrainset() - Method in class test.ml2db.al.utility.globaldistance.DistancesStoreTest


getPredictedBipartitions() - Method in class main.ml2db.mulan.measuremetrics.AnnotationsReader


getPredictedMultiLabelOutput(String) - Method in class main.ml2db.mulan.measuremetrics.AnnotationsReader
:   Get the predicted labels for that instance in MultiLabelOutput format

getPredictionsAndTruthReader() - Static method in class test.ml2db.mulan.measuremetrics.PredictionsPerformanceMeasurerTest


getPredictionsFilePath() - Method in class main.ml2db.mulan.measuremetrics.AnnotationsReader


getPredictionsInstancesAndLabels() - Method in class main.ml2db.mulan.measuremetrics.AnnotationsReader


getPredictionsTable() - Method in class main.ml2db.mulan.predict.PredictionsSaver


getPredRecordsFromDb() - Method in class main.ml2db.mulan.predict.PredictionsSaver
:   Get prediction records from db

getPredSaver() - Method in class main.ml2db.mulan.learn.traintest.TrainTestEvaluator


getProperties() - Method in class main.ml2db.weka.ArffProps
:   Get the properties

getProperties() - Method in class main.ml2db.weka.DataSetChecker


getPropertiesFilePath() - Method in class main.ml2db.weka.ArffPropsFromFile
:   Get the properties file path

getPropertiesFromFileFromDb(int) - Method in class main.ml2db.weka.ArffRecordsTable


getProperty(String) - Method in class main.ml2db.weka.ArffProps
:   Get a property value

getPropertyValueFromTable(String, int) - Method in class main.ml2db.weka.ArffRecordsTable


getQueries() - Method in class main.ml2db.al.utility.pickers.RandomPicker


getQuery(IdMultiLabelInstances) - Method in class main.ml2db.al.utility.pickers.RandomPicker


getRakel\_J48\_C45() - Static method in class main.ml2db.mulan.learn.MulanLearners
:   From Wikipedia: C4.5 is an algorithm used to generate a decision tree
    developed by Ross Quinlan.

getRakelWrappedClassifier(MultiLabelLearner, String) - Static method in class main.ml2db.mulan.learn.MulanLearners
:   RAKEL-MLKNN (RAndom k-labELsets) // failed not enough labels in metadata

getRandomAttPickerAL() - Static method in class test.ml2db.al.utility.pickers.RandomAttributePickerTest


getReader() - Method in class main.ml2db.mulan.measuremetrics.PredictionsPerformanceMeasurer


getReader() - Method in class main.ml2db.mulan.mulanarff.database.MulanArffRecord


getReader() - Static method in class test.ml2db.weka.ArffPropsTableTest


getRecord(int) - Static method in class test.ml2db.mulan.mulanarff.database.MulanArffRecordTest


getRemoteDbManagers() - Static method in class main.ml2db.ProjectParameters


getResults() - Method in class main.ml2db.mulan.measuremetrics.PredictionsPerformanceMeasurer


getResultsDbManager() - Method in class main.ml2db.al.learning.ExperimentDetails


getResultsDbManager() - Method in class main.ml2db.weka.Arff


getResultsDirectory() - Method in class main.ml2db.mulan.learn.crosseval.CrossOutputManager


getResultsFilePath() - Method in class main.ml2db.mulan.measuremetrics.PredictionsPerformanceMeasurer


getRevision() - Method in class main.ml2db.weka.JaccardDistance


getRowValues(Object[]) - Method in class main.ml2db.al.learning.AlOutputManager


getSavedPredictionsToString(TreeMap<String, int[]>) - Method in class main.ml2db.mulan.predict.PredictionsSaver
:   For test: load saved predictions from database for inspection.

getSaver(String, String, String, String, MulanLearner) - Static method in class test.ml2db.mulan.predict.PredictionsSaverTest


getSerialisationFilePath() - Static method in class main.ml2db.al.utility.pickers.byfrequency.NearestNeighboursByFreqUtility


getStartTime() - Method in class main.ml2db.al.ActiveLearning


getStartTime() - Method in class main.ml2db.mulan.learn.evaluation.EvaluatorNoAUC


getStartTime() - Method in class main.ml2db.mulan.predict.PredictionsSaver


getStatistics() - Static method in class test.ml2db.al.StatisticsTest


getStats(IdMultiLabelInstances) - Static method in class test.ml2db.mulan.mulanarff.StatisticsTest


getStatsTestArff() - Static method in class test.ml2db.mulan.mulanarff.StatisticsTest


getStatsTrainArff() - Static method in class test.ml2db.mulan.mulanarff.StatisticsTest


getStep() - Method in class main.ml2db.al.utility.pickers.RandomPicker


getStepId() - Method in class main.ml2db.al.ActiveLearning


getStepLimit() - Method in class main.ml2db.al.ActiveLearning


getStorage() - Method in class main.ml2db.al.utility.globaldistance.DistancesCalculator


getStore() - Method in class main.ml2db.al.utility.confidence.ConfidenceCalculator


getStoredFeatureAttributes() - Method in class main.ml2db.mulan.mulanarff.IdMultiLabelInstances


getStrictness() - Static method in class main.ml2db.mulan.learn.evaluation.EvaluatorNoAUC


getString() - Method in class main.ml2db.mulan.mulanarff.IdInstance


getStringPool() - Method in class main.ml2db.al.utility.globaldistance.DistancesCalculator


getTable() - Method in class main.ml2db.al.learning.AlOutputManager


getTable() - Method in class main.ml2db.mulan.learn.crosseval.CrossOutputManager
:   public TableRow getRowValues(Object[] results) { String run = (String)
    results[0]; int trainArffId = this.getExperimenter().getInputManager()
    .getTrainArffRecord().getArffId();
    // for crossevaluation, same id for training and testing int testArffId =
    this.getTestId(); String startOfRun = (String) results[1]; String
    endOfRun = (String) results[2]; MulanLearner learnerName =
    this.getExperimenter().getLearner(); Evaluation evaluationMetrics =
    (Evaluation) results[3];
    return EvaluationResultsRecord.createEvaluationRecord(run, startOfRun,
    endOfRun, learnerName, evaluationMetrics, Integer.toString(trainArffId),
    Integer.toString(testArffId),
    this.getTable().generateTableManager(this.getExperimentDbManager())); }

getTable() - Method in class test.ml2db.dataharness.DataTableTest


getTableName() - Method in class test.ml2db.dataharness.DataTableTest


getTableReader() - Method in class test.ml2db.dataharness.DataTableTest


getTableRow() - Method in class main.ml2db.al.learning.ExperimentDetails


getTechnicalInformation() - Method in class main.ml2db.mulan.classifier.lazy.BRkNNVisible


getTempFile() - Method in class main.ml2db.weka.DataSetGenerator


getTestArffId() - Method in class main.ml2db.mulan.learn.crosseval.CrossExperimenter


getTestArffId() - Method in class main.ml2db.mulan.predict.PredictionsSaver


getTestArffProps1() - Static method in class test.ml2db.weka.ArffTest


getTestArffProps2() - Static method in class test.ml2db.weka.ArffTest


getTestArffRecord() - Method in class main.ml2db.mulan.learn.traintest.TrainTestInputManager


getTestArffRecordsDbManager() - Static method in class test.ml2db.dataharness.TestProjectParameters


getTestDataDbManager() - Static method in class test.ml2db.dataharness.TestProjectParameters


getTestDataSet() - Method in class main.ml2db.mulan.learn.traintest.TrainTestInputManager


getTestDbManager() - Static method in class test.ml2db.alltests.DbManagerForTest


getTestDbManagers() - Static method in class test.ml2db.alltests.DbManagerForTest


getTestMachineLearningResultsDbManager() - Static method in class test.ml2db.dataharness.TestProjectParameters


getTestNeighboursPrinter() - Static method in class main.ml2db.mulan.mulanarff.NeighboursPrinterTest
:   PRINTING ONLY ! .

getTestSet() - Method in class main.ml2db.mulan.predict.PredictionsSaver


getToBePredicted() - Method in class main.ml2db.mulan.mulanarff.IdMultiLabelInstances


getTrainArff() - Static method in class test.ml2db.weka.ArffPropsTableTest


getTrainArffId() - Method in class main.ml2db.mulan.learn.crosseval.CrossExperimenter


getTrainArffId() - Method in class main.ml2db.mulan.predict.PredictionsSaver


getTrainArffPath() - Method in class main.ml2db.mulan.learn.crosseval.CrossInputManager


getTrainArffRecord() - Method in class main.ml2db.mulan.learn.crosseval.CrossInputManager


getTrainAttributesAndTrainTestClasses() - Method in class main.ml2db.mulan.attributesfiltered.AttFilter


getTrainDataSet() - Method in class main.ml2db.mulan.learn.crosseval.CrossInputManager


getTrainedLearner() - Method in class main.ml2db.al.ActiveLearning


getTrainedLearner() - Method in class main.ml2db.mulan.predict.PredictionsSaver


getTrainedLearner(IdMultiLabelInstances) - Static method in class test.ml2db.al.utility.confidence.ConfidenceCalculatorTest


getTrainedLearner1() - Static method in class test.ml2db.al.utility.confidence.ConfidenceCalculatorTest


getTrainedLearner2() - Static method in class test.ml2db.al.utility.confidence.ConfidenceCalculatorTest


getTrainedModelFile() - Method in class main.ml2db.mulan.learn.traintest.TrainTestEvaluatorSerialised


getTrainedModelFile() - Method in class main.ml2db.mulan.learn.traintest.TrainTestExperimenterSerialized


getTrainNeighboursPrinter() - Static method in class main.ml2db.mulan.mulanarff.NeighboursPrinterTest


getTrainSet() - Method in class main.ml2db.mulan.predict.PredictionsSaver


getTrainsetString() - Static method in class test.ml2db.dataharness.LoadFirstValidArff


getTrainTestFolds(int) - Method in class main.ml2db.mulan.learn.crosseval.Folds
:   Get the train and test data for crossevaluation fold i

getTrainTestFolds(int) - Method in class main.ml2db.mulan.learn.crosseval.StratifiedFolds


getTrainXmlPath() - Method in class main.ml2db.mulan.attributesfiltered.AttFilter


getTrainXmlPath() - Method in class main.ml2db.mulan.learn.crosseval.CrossInputManager


getTrueLabels(IdInstance) - Method in class main.ml2db.mulan.mulanarff.IdMultiLabelInstances


getTruePositives() - Method in class main.ml2db.mulan.learn.evaluation.MicroFMeasurePrinter


getTruthBipartition(String) - Method in class main.ml2db.mulan.measuremetrics.AnnotationsReader


getTruthBipartitions() - Method in class main.ml2db.mulan.measuremetrics.AnnotationsReader


getTruthFilePath() - Method in class main.ml2db.mulan.measuremetrics.AnnotationsReader


getTruthInstancesAndLabels() - Method in class main.ml2db.mulan.measuremetrics.AnnotationsReader


getUncertainty(MultiLabelLearner, IdInstance) - Method in class main.ml2db.al.utility.confidence.ConfidenceCalculator
:   For each confidences set (double[]), calculate the uncertainty and store
    it.

getUncertaintyValue(double) - Static method in class main.ml2db.al.utility.confidence.ConfidenceCalculator
:   // the confidence certainty decreases the closer it is to 0.5 // (e.g.

getUndirectedGraphvizString(IdInstance) - Static method in class main.ml2db.graphviz.GraphvizInstance


getUnion(TreeSet<Integer>, TreeSet<Integer>) - Method in class main.ml2db.weka.JaccardDistance
:   Calculate the size of the union of set A (feature attributes of instance
    A) and set B (feature attributes of instance B), that is, all the
    non-zero attributes the two instances have.

getUnion(TreeSet<Integer>, TreeSet<Integer>) - Method in class main.ml2db.weka.JaccardDistanceStored
:   Calculate the size of the union of set A (feature attributes of instance
    A) and set B (feature attributes of instance B), that is, all the
    non-zero attributes the two instances have.

getUnlabelled() - Method in class main.ml2db.al.ActiveLearning


getUnlabelled() - Method in class main.ml2db.al.utility.UtilityMetric


getUnlabelledArffId() - Method in class main.ml2db.al.learning.ExperimentDetails


getUnlabelledDistances(Instance) - Method in class main.ml2db.al.utility.globaldistance.GlobalDistanceMetric


getUnlabelledInstances() - Method in class main.ml2db.al.learning.ExperimentDetails


getUnlabelledInstances() - Method in class main.ml2db.al.utility.neighbours.NeighbourhoodNetwork


getUnlabInstances() - Method in class main.ml2db.al.learning.ExperimentDetails


getUtility(MultiLabelLearner, IdInstance) - Method in class main.ml2db.al.utility.confidence.ConfidenceMetric
:   Gets a measure of how distant the instance is from other unlabelled
    instances (good if it's close to them) and labelled instances (bad if
    it's close to them) and multiplies it by the average confidence certainty
    (see Confidence class).

getUtility(MultiLabelLearner, IdInstance) - Method in class main.ml2db.al.utility.globaldistance.GlobalDistanceMetric
:   Gets a measure of how distant the instance is from other unlabelled
    instances (good if it's close to them) and labelled instances (bad if
    it's close to them).

getUtility(MultiLabelLearner, IdInstance) - Method in class main.ml2db.al.utility.hybrid.GlobalDistanceWithConfidenceMetric
:   Gets a measure of how distant the instance is from other unlabelled
    instances (good if it's close to them) and labelled instances (bad if
    it's close to them) and multiplies it by the average confidence certainty
    (see Confidence class).

getUtility(MultiLabelLearner, IdInstance) - Method in class main.ml2db.al.utility.neighbours.NeighbourhoodNetwork
:   Only works with identified instances.

getUtility(MultiLabelLearner, IdInstance) - Method in class main.ml2db.al.utility.pickers.RandomPicker


getUtility(MultiLabelLearner, IdInstance) - Method in class main.ml2db.al.utility.UtilityMetric


getUtilityMetric() - Method in class main.ml2db.al.learning.ExperimentDetails


getUtilityTableRowSaver() - Method in class main.ml2db.al.database.AlDataSaver


getValue() - Method in class main.ml2db.mulan.learn.evaluation.FalseNegatives


getValue() - Method in class main.ml2db.mulan.learn.evaluation.FalsePositives


getValue() - Method in class main.ml2db.mulan.learn.evaluation.MicroFMeasurePrinter


getValue() - Method in class main.ml2db.mulan.learn.evaluation.TruePositives


getValueOptions() - Method in class test.ml2db.weka.AttributeFactoryTest


getVector() - Method in class main.ml2db.mulan.mulanarff.IdMultiLabelInstances


getWekaInst2IdInstMap() - Method in class main.ml2db.mulan.mulanarff.IdMultiLabelInstances


getWekaInstance() - Method in class main.ml2db.mulan.mulanarff.IdInstance


getWrittenMulan(MulanArff) - Static method in class test.ml2db.mulan.mulanarff.MulanArffTest


getXmlFileName() - Method in class main.ml2db.mulan.mulanarff.database.MulanArffRecord


getXmlFilePath() - Method in class main.ml2db.mulan.mulanarff.IdMultiLabelInstances


getXmlFilePath() - Method in class main.ml2db.mulan.mulanarff.ImportMulanArff


getXmlFilePath() - Method in class main.ml2db.weka.ArffProps
:   Get the path to the Mulan XML file

getXmlPathById(int) - Method in class main.ml2db.mulan.attributesfiltered.AttFilter


getXMLpathFromArffPath(String) - Static method in class main.ml2db.mulan.mulanarff.MulanArff
:   Generates the xml file path: same name+path as .arff file, but .xml
    termination

getXmlQueryById(int) - Method in class main.ml2db.mulan.attributesfiltered.AttFilter


getZeroRule() - Static method in class main.ml2db.mulan.learn.MulanLearners
:   public static MulanLearner
    getWrappedClassifier(TransformationBasedMultiLabelLearner wrapper, String
    wrapperName, Classifier classifier, String classifierName){
    TransformationBasedMultiLabelLearner wrapped = new
    TransformationBasedMultiLabelLearner(classifier); wrapper.setDebug(true);
    return new MulanLearner(MulanLearner.getName(wrapper), wrapperName + "\_"
    + classifierName, wrapped); }

getZeros() - Method in class main.ml2db.weka.AttributeFactory


GIF - Static variable in class main.ml2db.graphviz.GraphViz


GlobalDistanceMetric - Class in main.ml2db.al.utility.globaldistance
:   Positioning metric: a metric that considers positively if an instance is
    close to other unlabelled instances and distant from other labelled
    instances.

GlobalDistanceMetric() - Constructor for class main.ml2db.al.utility.globaldistance.GlobalDistanceMetric


GlobalDistanceMetricTest - Class in test.ml2db.al.utility.globaldistance
:   Class

GlobalDistanceMetricTest() - Constructor for class test.ml2db.al.utility.globaldistance.GlobalDistanceMetricTest


GlobalDistanceWithConfidenceMetric - Class in main.ml2db.al.utility.hybrid
:   Combines a Positioning metric (a metric that considers positively if an
    instance is close to other unlabelled instances and distant from other
    labelled instances) with the average certainty of the instance prediction
    (that is, how distant the average of confidence over all labels is from 50%)

GlobalDistanceWithConfidenceMetric() - Constructor for class main.ml2db.al.utility.hybrid.GlobalDistanceWithConfidenceMetric


GlobalDistanceWithConfidenceMetricTest - Class in test.ml2db.al.utility.hybrid
:   Class

GlobalDistanceWithConfidenceMetricTest() - Constructor for class test.ml2db.al.utility.hybrid.GlobalDistanceWithConfidenceMetricTest


globalInfo() - Method in class main.ml2db.mulan.classifier.lazy.BRkNNVisible


globalInfo() - Method in class main.ml2db.weka.JaccardDistance


GlobalPositionStore - Class in main.ml2db.al.utility.globaldistance
:   Stores (and updates) a map containing as key one (kind of/set of attributes)
    instance and as value the number representing the distance between the
    instance and a set of other labelled or unlabelled instances.

GlobalPositionStore(int, GlobalDistanceMetric) - Constructor for class main.ml2db.al.utility.globaldistance.GlobalPositionStore


GraphViz - Class in main.ml2db.graphviz
:   Purpose: GraphViz Java API
    Description:
    With this Java class you can simply call dot from your Java programs
    Example usage:

GraphViz() - Constructor for class main.ml2db.graphviz.GraphViz
:   Constructor: creates a new GraphViz object that will contain a graph.

GraphViz(String) - Constructor for class main.ml2db.graphviz.GraphViz
:   Constructor: creates a new GraphViz object that will contain a graph.

GRAPHVIZ\_HEADER - Static variable in class main.ml2db.mulan.mulanarff.NeighboursPrinter


GraphVizArff - Class in main.ml2db.graphviz
:   Generates a GraphViz representation (.dot file) of the instances, class
    labels and attributes in a Weka/Mulan dataset and their relations.

GraphVizArff(String) - Constructor for class main.ml2db.graphviz.GraphVizArff


GraphVizArffColoured - Class in main.ml2db.graphviz
:   Nodes and edges can of a base dot file can be coloured to show their labelled
    status (train set), or their correctly/uncorrectly predicted status (test
    set).

GraphVizArffColoured(String) - Constructor for class main.ml2db.graphviz.GraphVizArffColoured


GraphVizArffColouredTest - Class in test.ml2db.graphviz
:   Class

GraphVizArffColouredTest() - Constructor for class test.ml2db.graphviz.GraphVizArffColouredTest


GraphVizArffTest - Class in test.ml2db.graphviz
:   Class

GraphVizArffTest() - Constructor for class test.ml2db.graphviz.GraphVizArffTest


GraphvizInstance - Class in main.ml2db.graphviz
:   Class

GraphvizInstance() - Constructor for class main.ml2db.graphviz.GraphvizInstance


GraphVizPrinter - Class in main.ml2db.graphviz
:   Class

GraphVizPrinter() - Constructor for class main.ml2db.graphviz.GraphVizPrinter


GraphVizPrinterTest - Class in test.ml2db.al
:   Class

GraphVizPrinterTest() - Constructor for class test.ml2db.al.GraphVizPrinterTest


GraphVizTest - Class in test.ml2db.graphviz
:   Class

GraphVizTest() - Constructor for class test.ml2db.graphviz.GraphVizTest


guessNeedsNominalOrNumeric() - Method in class main.ml2db.weka.InstAttValueMap

A B C D E F G H I J K L M N O P Q R S T U V W X

- Overview
- Package
- Class
- Use
- Tree
- Deprecated
- Index
- Help

- Prev Letter
- Next Letter

- Frames
- No Frames

- All Classes
